# Magnitude and determinants of abnormal uterine bleeding among reproductive-age women in Kombolcha Rejiopolitan Administrative Town, Northeast Ethiopia: a FIGO-based study (2023)

**DOI:** 10.3389/frph.2025.1559105

**Published:** 2025-04-16

**Authors:** Abubeker Seid, Gizachew Abdissa Bulto, Adem Yesuf, Ali Yimer, Hassen Ahmed, Tsegaye Benti Muse

**Affiliations:** ^1^ Department of Nursing, College of Health Sciences, Woldia University, Woldia, Ethiopia; ^2^ Department of Midwifery, College of Health Sciences and Referral Hospital, Ambo University, Ambo, Ethiopia; ^3^ Department of Midwifery, College of Health Sciences, Woldia University, Woldia, Ethiopia; ^4^ Department of Public Health, College of Health Sciences, Woldia University, Woldia, Ethiopia; ^5^ Department of Biomedical Science, College of Health Sciences, Woldia University, Woldia, Ethiopia; ^6^ Department of Public Health, College of Health Sciences and Referral Hospital, Ambo University, Ambo, Ethiopia

**Keywords:** abnormal uterine bleeding, reproductive-age women, Kombolcha, Ethiopia, AUB

## Abstract

**Background:**

Abnormal uterine bleeding (AUB) is a common reason for women to seek healthcare, affecting their health, quality of life, productivity, and increasing the risk of complications. While menstrual disorders have been studied in Ethiopia, the prevalence and determinants of AUB among reproductive-age women remain poorly understood due to the use of outdated terminology and a lack of standardized approaches, which hinder accurate assessment and comparison with international findings. This study aims to address this gap by assessing the magnitude and determinants of AUB among reproductive-age women in Kombolcha town, Ethiopia, using the 2018 FIGO criteria.

**Methods:**

A community-based cross-sectional study was conducted among 608 reproductive-age women in Kombolcha town from April 20 to May 20, 2023, using systematic random sampling. Data were collected using interviewer-administered structured questionnaires. The data were entered into EPI Data version 4.6 and analyzed using SPSS version 26. All predictor variables with a *p*-value <0.25 in bivariable analysis were included in multivariable logistic regression. Variables with a *p*-value <0.05 in the multivariable analysis were considered statistically significant predictors of abnormal uterine bleeding.

**Result:**

This study found that 36.5% of 591 reproductive-age women experienced abnormal uterine bleeding (AUB). Several factors were significantly associated with AUB: Women in late reproductive age (41–49 years) had 4.2 times higher odds of experiencing AUB compared to those in mid-reproductive age (AOR = 4.181, 95% CI: 2.199–7.948). Hypertensive women were 3.71 times more likely to experience AUB (AOR = 3.706, 95% CI: 1.106–12.417). Women with a history of abortion had 2.3 times higher odds of AUB (AOR = 2.345, 95% CI: 1.069–5.147). A history of diagnosed anemia and late menarche (≥15 years) were also significantly associated with increased AUB risk (AOR = 2.939, 95% CI: 1.437–6.010; AOR = 3.824, 95% CI: 2.199–7.948).

**Conclusion:**

This study highlights the significant burden of AUB, with 36.5% of women affected. Healthcare providers should educate patients about lifestyle changes, treatment options, and when to seek emergency care for AUB to improve health outcomes.

## Background

Abnormal uterine bleeding (AUB) is defined as “menstrual pattern bleeding that is abnormal in frequency (cycle length <24 days or >38 days), duration of flow (>8 days), volume of flow (light or heavy), or regularity (shortest to longest cycle variation ≥ 8–10 days) in a woman who is not pregnant (1).” Prior to the last ten years, many confusing terms had been used to refer to abnormal uterine bleeding. The International Federation of Obstetrics and Gynecology (FIGO) has established a new definition and a standardized classification system for abnormal uterine bleeding (1, 2). Although Heavy Menstrual Bleeding (HMB) is currently classified as a subset of abnormal uterine bleeding (AUB), the prior definition of heavy menstrual bleeding (HMB) was replaced by the more patient-centered definition proposed by the National Institute for Care and Excellence with “excessive menstrual loss that interferes with the physical, social, emotional, or maternal quality of life (3).”

Abnormal uterine bleeding is a prevalent issue that affects women's quality of life, productivity, and healthcare costs (4, 5). If left untreated, it may be associated with complications such as infertility, endometrial cancer, severe anemia, hypotension, shock, and even death (1). AUB has also been linked to an increased risk of premature mortality due to cardiovascular and noncommunicable diseases, including ovarian cancer, type 2 diabetes, and mental health problems (6).

Epidemiological studies have suggested that approximately 10% to 30% of reproductive-aged women are affected by heavy menstrual bleeding. However, the prevalence of AUB, a broader clinical condition than HMB, could be higher than the estimated 10% to 30% (7). The prevalence of AUB ranged from 38.3% to 77.6% in southern Asia and sub-Saharan Africa, with a pooled average of 48.6% (8).

In Ethiopia, most published studies conducted on menstrual disorders have shown the magnitude of dysmenorrhea, premenstrual symptoms, and menstrual irregularity. However, none of them studied in general the magnitude and factors associated with AUB among reproductive-age women, except the study conducted in Jimma (4, 9, 10). Although a study was conducted in Debre-Berhan, it was institutional, focused on the same age group, and defined menstrual irregularity as abnormal uterine bleeding. Additionally, the study conducted in Jimma does not clearly define abnormal uterine bleeding and uses the terms metrorrhagia, menorrhagia, polymenorrhea, and oligomenorrhea, which have been replaced by FIGO with terms that are universally understood. Therefore, considering the inconsistency in previous studies and the lack of adequate published research in Ethiopia, this research aimed to assess the magnitude and associated factors of abnormal uterine bleeding among women of reproductive age by using the revised 2018 FIGO recommendations on terminologies and definitions for normal and abnormal uterine bleeding (1).

## Methods and materials

### Study area, period and design

A community-based cross-sectional study was employed from April 20 to May 20, 2023, in Kombolcha Town, Northeast Ethiopia. The town is found at a latitude and longitude of 11°5′N 39°44′E/11.083° N 39.733°E with an elevation between 1,842 and 1,915 meters above sea level. It is situated 379 km from Addis Ababa and 498 km from Bahir-Dar City, the capital of the Amhara regional state. The town has one referral hospital, four health centers, and twenty health posts. It has 20 Kebeles (14 urban and 6 rural Kebeles) with a total population of 175,767, of whom 84,717 are men and 91,056 women. The total number of households and women of reproductive age is 40,876 and 41,446, respectively (11).

### Source and study population

All women of reproductive age (15–49) who were living in the selected Kebeles of Kombolcha Rejiopolitan Administrative Town were the source population, whereas all women of reproductive age (15–49 years old) who were living in the selected Kebeles of Kombolcha Town were the study population.

### Inclusion criteria

Women of reproductive age (15–49 years old) who were living in Kombolcha Rejiopolitan Administrative Town for at least 6 months were included.

### Exclusion criteria

Women of reproductive age who were seriously ill, in early menopause, pregnant, and breastfeeding were excluded.

### Sample size determination and sampling procedure

The sample size was calculated for each specific objective, and then the largest sample size was taken as a final sample size. The sample size for the first specific objective was determined by using a single population proportion formula by considering the following assumptions: *p* = 34.1% [prevalence of AUB in Jimma, Oromia, Ethiopia (12), 95% level of confidence, and 5% margin of error]. Thus, *n* = [(Z*α*/2)2 * *p* (1−*p*)]/d2 = 346. For the second objective (which was to identify factors), the sample size was calculated using Epi-info version 7 by considering the following assumptions: 95% confidence interval, 80% power, ratio of unexposed to exposed 1:1, AOR, and adding proportion of variable among unexposed and exposed, which are taken from previous studies for each variable. Hence, the largest sample size was *n* = 552, and after adding a 10% nonresponse rate, the final sample size became *n* = 607.2 = 608.

Seven Kebeles were randomly selected by the lottery method from 20 Kebeles of Kombolcha Town. The final sample size was proportionally allocated to each Kebele based on the number of households with reproductive-age women. A systematic random sampling technique was used to select the participants. The interval of the sample was *k* = 18, and it was determined based on the total number of households with reproductive-age women of each Kebele. Then, the first household was randomly selected by the lottery method between 1 and 18, and it was seven. Then, every 18th household with reproductive-age women was selected. When more than one eligible woman was available in the selected household, a simple random sampling method (lottery method) was employed.

### Data collection instruments and procedures

The data were collected through interviews using the pre-tested, structured Amharic version questionnaire. In addition, anthropometric measurements (height and weight) were performed to calculate the body mass index (BMI) of the participants. The questionnaire includes sociodemographic data, menstruation-related questions, lifestyle and behavioral questions, medical history questions and anthropometric measurements. After analyzing several works in the literature, the questionnaire was first written in English, then translated into Amharic, and then back to English. A person who was an expert in both languages checked the questionnaires’ consistency. In addition, a pretest was conducted with 5% (31) of the sample size on reproductive-age women in the Harbu Administrative Town to check the soundness of the data collection tool. At the end of the pretest, it was found that the instruments needed further correction and refining in demographic data and logical flow of questions. Finally, the investigator made corrections.

### Study variables and measurements

The dependent variable was abnormal uterine bleeding. The International Federation of Gynecology and Obstetrics (FIGO) 2018 criteria for abnormal uterine bleeding definition was used to establish whether the uterine bleeding was normal or abnormal. As a result, in the current study, abnormal uterine bleeding was considered if at least one of the following menstrual pattern bleeding was complained of by the woman: a frequency (cycle length) of <24 or >38 days, a duration of bleeding exceeding 8 days, an individual assessment of the quantity (heavy or light), and a shortest to longest cycle variance of ≥8–10 days (slight variation with age, (18–25 years ≥10 days; 26–41 years ≥8 days; 42–49 years ≥10 days) during the previous 3 months (1).

Intermenstrual bleeding: irregular episodes of bleeding occurring between otherwise fairly normal menstrual periods (1).

Heavy menstrual bleeding: This is based on a complaint by the woman; excessive blood loss, which interferes with the woman's physical, emotional, social, and maternal quality of life (1).

Sociodemographic factors: age, educational status, occupation, family monthly income level, and marital status.

Lifestyle-related factors; (underweight, normal, overweight/obese), alcohol consumption, and cigarette smoking.

Menstrual and reproductive-related factors: parity, age at menarche, IUCD usage, history of hormonal contraceptive use, and history of abortion (any pregnancy loss before 28 weeks of gestation, as per the Ethiopian Ministry of Health guidelines).

Chronic and history of underlying disease factors: history of uterine fibroids, history of STIs, bleeding disorder, history of diagnosed anemia, hypertension, diabetes mellitus and thyroid disorder.

Alcohol consumption is categorized as nondrinker, occasional (drinking alcohol less than one time per week), and regular (drinking alcohol at least one time per week) (12, 13).

Body mass index (BMI): Based on the calculated BMI, the study participants were classified as underweight (BMI < 18.5), normal weight (BMI = 18.5–24.9), overweight (BMI = 25–29.9), and obese (BMI ≥ 30) (14).

Smoking status is divided into three categories: nonsmoker, occasional (smokes, but not every day), and regular (at least one cigarette per day for at least six consecutive months) (12, 13).

### Data analysis

Data entry was performed using EPI-Data version 4.6, and the dataset was exported to SPSS version 26 for analysis. Normality tests, model fit assessments, multicollinearity checks, and tests for homogeneity of variance were performed. Descriptive statistics, including frequencies and percentages, were used for categorical variables, while continuous variables were presented as means ± standard deviations. Independent variables with a *p*-value less than 0.25 in the bivariable analysis were considered for inclusion in the final model. These variables were then analyzed using multivariable binary logistic regression (a backward regression analysis) to identify significant factors and control for potential confounders. Multivariable adjusted odds ratios, along with 95% confidence intervals, were calculated, and statistical significance was determined at a *p*-value of less than 0.05. Hosmer and Lemeshow's goodness-of-fit test indicated that the assumptions for multiple logistic regression were met, with a *p*-value of 0.817 and a chi-square value of 3.672.

## Results

### Sociodemographic characteristics of participants

A total of 591 reproductive-age women participated in this study, yielding a response rate of 97.2%. The mean (±SD) age of the participants was 32.42 (±8.26) years. The majority of respondents (83.9%, *n* = 496) were married. Regarding educational attainment, 215 participants (36.4%) had completed primary education, while 140 participants (23.7%) had no formal education. The mean (±SD) monthly income of the respondents was 2,647 (±2,071) ETB (Table 1).

**Table 1:** Sociodemographic characteristics of reproductive age women in Kombolcha town, Amhara, Ethiopia, (*n* = 591).

Variable	Category	Frequency	Percentage (%)
Age	15–19	47	8.0
	20–40	422	71.4
	41–49	122	20.6
Marital status	Single	64	10.8
	Married	496	83.9
	Divorce	21	3.6
	Widowed	10	1.7
Educational status	Had no formal education	140	23.7
	Primary school	215	36.4
	Secondary and above	236	39.9
Occupational status	House wife	357	60.4
	Daily laborer	18	3.0
	Merchant	50	8.5
	Student	66	11.2
	Employee	100	16.9
Income (ETB)	<1,000	81	13.7
	1,000–1,999	192	32.5
	2,000–2,999	165	27.9
	≥3,000	153	25.9

### Reproductive and lifestyle-related characteristics of participants

The mean age at menarche was 13.94 ± 1.269 years, with a range of 11–17 years. Over two-thirds (67.5%) of the participants were multiparous. Approximately 12.9% (*n* = 76) of participants had a history of at least one abortion. More than three-quarters (76.8%) of the participants had a normal body mass index (BMI). Regular alcohol consumption was reported by 2.9% (*n* = 70) of the respondents, and only 1.4% (*n* = 8) of participants were occasional tobacco smokers (Table 2).

**Table 2:** Reproductive history and lifestyle-related characteristics of reproductive-age women in kombolcha town, amhara, Ethiopia (*n* = 591).

Variable	Category	Frequency(n)	Percentage (%)
Hormonal contraceptive use	Yes	302	51.1
	No	289	48.9
History of abortion	Yes	76	12.9
	No	515	87.1
History of IUCD use	Yes	21	3.6
	No	570	96.4
Menarcheal-age	Early	74	12.5
	Average/Normal	320	54.1
	Delayed	197	33.3
Parity	Nulli-para	88	14.9
	Primi-para	49	8.3
	Multi-para	399	67.5
	Grand Multipara	55	9.3
Body mass index (BMI)	Under weight	24	4.1
	Normal	454	76.8
	Overweight	113	19.1
Alcohol consumption	Never	523	88.5
	Occasionally	51	8.6
	Regularly	17	2.9
Tobacco Smoking	Occasionally	8	1.4
	Never	583	98.6

### History of chronic and underlying disease of the study participants

This study found that 13.9% (*n* = 82) of participants had a history of anemia, 6.3% had hypertension, and 5.4% had been diagnosed with diabetes by healthcare professionals at health facilities (Table 3).

**Table 3:** History of chronic and underlying disease of women in the reproductive-age group in Kombolcha town, Ethiopia, 2023 (*n* = 591).

Variable	Category	Frequency (*n*)	Percentage (%)
Hypertension^a^	Yes	37	6.3
	No	554	93.7
Diabetes^a^	Yes	32	5.4
	No	559	94.6
STIs^a^	Yes	8	1.4
	No	583	98.6
Anemia^a^	Yes	82	13.9
	No	509	86.1
Thyroid Disorder^a^	Yes	12	2.0
	No	579	98.0

^a^ Self-reported (as diagnosed at health facilities by health professionals).

### Prevalence and pattern of abnormal uterine bleeding

**Figure 1:**
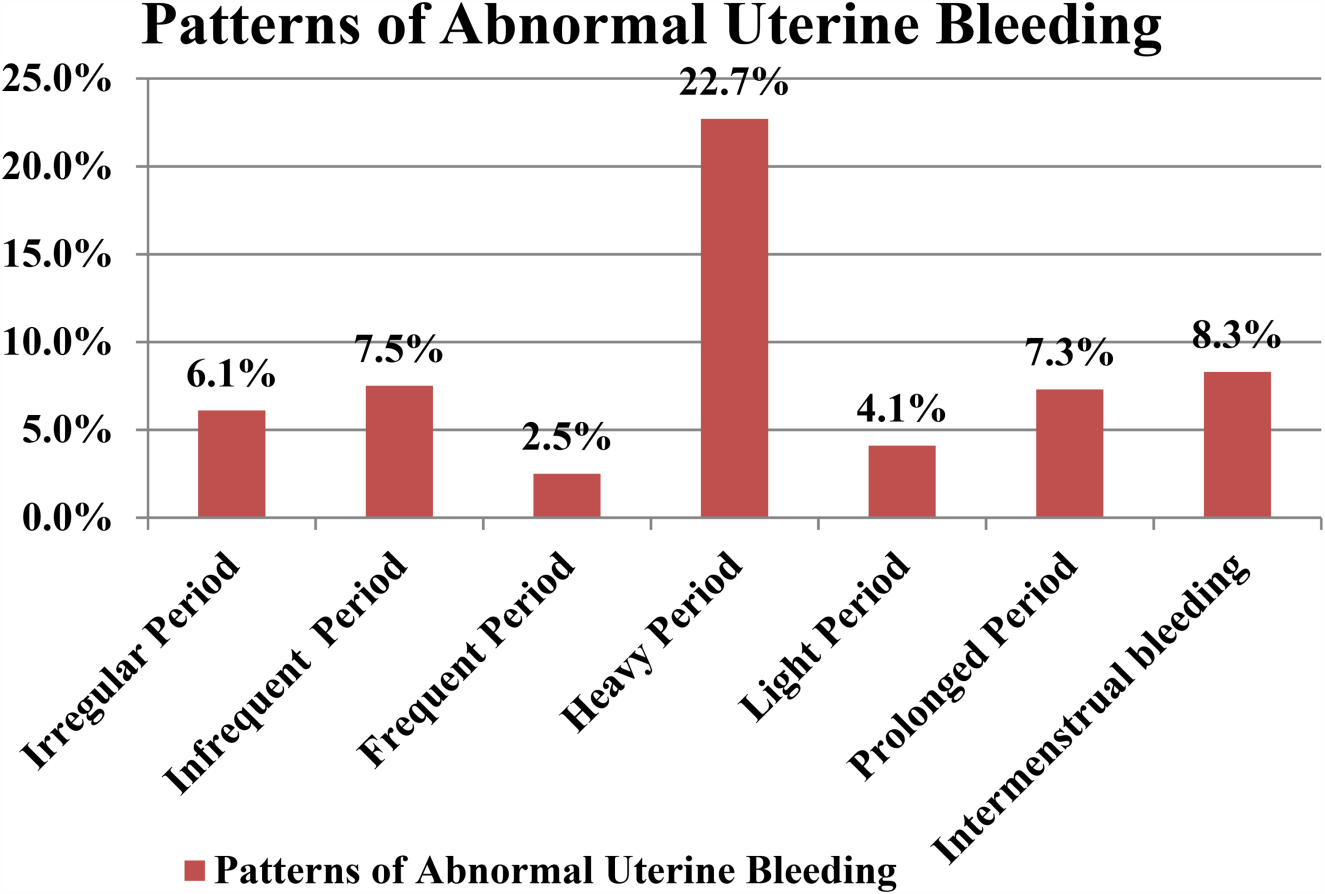
Patterns of abnormal uterine bleeding among reproductive age women in Kombolcha town, Ethiopia, 2023 (*n* = 591).

According to the FIGO definition and terminology for abnormal uterine bleeding (AUB), 214 participants (36.2%) [95% CI: 32.5–40.3] experienced abnormal uterine bleeding. The most prevalent form of AUB was heavy menstrual bleeding, which affected 22.7% of the women, while frequent menstrual bleeding was relatively rare, occurring in only 2.5% of participants. Notably, none of the respondents reported amenorrhea (Figure 1).

### Factors associated with abnormal uterine bleeding

In the bivariable logistic regression analysis, factors such as age, alcohol consumption, age at menarche, history of abortion, history of anemia, hypertension, diabetes mellitus, history of IUCD use, body mass index (BMI), and hormonal contraceptive use were found to be statistically associated with abnormal uterine bleeding (AUB), with statistical significance set at *p* < 0.25. In the multivariable logistic regression analysis, age (41–49 years), age at first menstruation (≥15 years), history of abortion, history of anemia, and hypertension were found to be independently associated with abnormal uterine bleeding.

The odds of abnormal uterine bleeding (AUB) were 4.2 times higher among women of late reproductive age (41–49 years) compared to those in the mid-reproductive age group (AOR = 4.181, 95% CI: 2.199–7.948). Women with hypertension were 3.71 times more likely to experience AUB than their non-hypertensive counterparts (AOR = 3.706, 95% CI: 1.106–12.417). Additionally, women with a history of abortions were 2.3 times more likely to experience AUB (AOR = 2.345, 95% CI: 1.069–5.147). There was also a significant association between a history of diagnosed anemia and an increased likelihood of AUB (AOR = 2.939, 95% CI: 1.437–6.010). Furthermore, women with late or delayed menarche (≥15 years) were 3.8 times more likely to experience AUB compared to those with early menarche (≤12 years) (AOR = 3.824, 95% CI: 2.199–7.948) (Table 4).

**Table 4:** Bivariable and multivariable logistic regression analysis of factors associated with abnormal uterine bleeding among women aged 15–49 years in Kombolcha town, Amhara, Ethiopia, 2023 (*n* = 591).

Variables	Category	AUB		Bivariable	Multivariable analysis	*p* value
		Yes	No	COR (95% CI)	AOR (95% CI)	
Age	15–19	10	37	0.739 (0.356–1.535)	2.109 (0.588–7.564)	0.252
	41–49	91	31	8.027 (5.062–12.729)	**4.181** (**2.199–7.948)**	<0.001
	20–40	113	309	1	1	
Alcohol consumption	Regular	7	10	1.334 (0.499–3.563	0.304 (0.069–1.334)	0.115
	Occasional	27	24	2.144 (1.202–3.824)	0.787 (0.355–1.748)	0.557
	Never	180	343	1	1	
Body Mass Index (BMI)	Underweight	6	18	0.788 (0.306–2.028)	4.330 (0.882–21.255)	0.071
	Overweight	73	40	4.312 (2.791–6.662)	1.814 (0.958 -3.435)	0.067
	Normal	135	319	1	1	
Age at menarche	13–14 years	17	297	0.051 (0.026–0.100	0.072 (0.033–0.159)	< 0.001
	≥15 years	158	45	3.151 (1.793–5.538)	**3.824** (**2.020–7.241)**	< 0.001
	≤12 years	39	35	1	1	
Hormonal contraceptive	Yes	97	205	0.696 (0.497–0.974)	1.311 (0.708–2.427)	0.388
	No	117	172	1	1	
History of IUCD use	Yes	16	5	6.012 (2.170–16.654)	4.859 (0.892–26.473)	0.068
	No	198	372	1	1	
History of Abortion	Yes	54	22	5.446 (3.207–9.250)	**2.345** (**1.069–5.147)**	0.034
	No	160	355	1	1	
History of Anemia	Yes	46	36	2.594 (1.615–4.165)	**2.939** (**1.437–6.010)**	0.003
	No	168	341	1	1	
Hypertension	Yes	26	11	4.602 (2.225–9.516)	**3.706** (**1.106–12.417)**	0.034
	No	188	366	1	1	
Diabetes mellitus	Yes	19	13	2.728 (1.319–5.642)	0.812 (0.278–2.369)	0.703
	No	195	364	1	1	

The bolded values are represent statistically significant associations within the corresponding confidence intervals (CIs).

## Discussion

According to this study, 214 women (36.2%) [95% CI: 32.5–40.3] had experienced abnormal uterine bleeding. This finding is consistent with studies conducted in Jimma (34.1%) (12), Debre-berhan (33.4%) (10), and Tehran (Iran) (35.8%) (4). However, it is lower than the prevalence reported in studies from Sudan (59.4%) (15), and Lebanon (80.7%) (16). The higher prevalence in Sudan and Lebanon may be attributed to the fact that these studies focused solely on university students aged 18–24 and 18–26, respectively, where the prevalence of menstrual irregularity tends to be higher (4). On the other hand, it might be related to the narrow range for the length of the cycle to consider normal menstruation (28–32 days) used by both studies, which could overestimate the prevalence. However, this study used the normal ranges for length of cycle as per FIGO definition (a frequency of 24–38 days). Additionally, the higher prevalence might be associated with the stress experienced by university students, which can have psychological effects that contribute to menstrual irregularities (10).

However, the prevalence in this study was higher than those reported in studies from Cameroon (3.7%) (17), China (18.2%) (13), Assam (India) (24.48%) (18), and Nepal (8.9%) (19). This discrepancy may be attributed to differences in study settings, classification methods, and definitions of abnormal uterine bleeding (AUB). The lower prevalence observed in the studies from Cameroon, India, and Nepal could be due to AUB being clinically diagnosed only when women presented with bleeding that deviated from the normal menstrual cycle. It is well documented that over 50% of women with AUB do not seek medical care, which could lead to an underestimation of AUB prevalence in these studies. Additionally, the Chinese study focused exclusively on heavy menstrual bleeding, potentially overlooking other patterns of AUB and further underestimating the overall prevalence of abnormal uterine bleeding.

The present study reveals a significant finding: the odds of abnormal uterine bleeding (AUB) among women in late reproductive age were 4.2 times higher than those in mid-reproductive age. This result aligns with a study conducted in Bihar, India, where the prevalence of menstrual disorders, including excessive bleeding, increased with age, particularly in women aged 40 and older (20). Similarly, research in Tehran, Iran, showed that the prevalence of AUB also rises in late reproductive ages, with nearly half of the women experiencing various types of AUB, especially heavy and prolonged menstruation (4). These associations can likely be attributed to the onset of the climacteric phase, during which menstrual cycles become irregular, often shortening or lengthening, and may become anovulatory. This is due to the decline in ovarian follicles and estradiol levels. As a result, there is a disruption in the hypothalamo-pituitary-ovarian axis, leading to an increase in serum FSH levels (21). Furthermore, perimenopausal women are at a higher risk of developing pelvic pathologies, such as fibroids and endometriosis, which can exacerbate bleeding and contribute to more severe menstrual problems (22).

In contrast, studies conducted in Debre Berhan and China found a significant association between early reproductive age and abnormal uterine bleeding (AUB) (10, 13). The discrepancy between these findings and the current study may be attributed to differences in the age groups used. Notably, the Debre Berhan study did not include women in late reproductive age, which may have limited the ability to accurately detect associations with AUB in this group. Additionally, the Chinese study focused solely on heavy menstrual bleeding (HMB), excluding other patterns of AUB. Previous research suggests that younger women are more likely to perceive moderate blood loss as very heavy compared to older women (23), which could lead to an overestimation of the prevalence of HMB among younger women.

This study also revealed a statistically significant association between the age at menarche and abnormal uterine bleeding (AUB). Women who experienced menarche at a late or delayed age (≥15 years) were 3.8 times more likely to develop AUB compared to those who had early menarche (≤12 years). This finding is consistent with a cross-sectional study conducted on Korean nurses, which found that a late age at menarche was associated with a higher likelihood of irregular menstruation (24). Similarly, a survey at Oita Medical University in Japan demonstrated that women with delayed menarche had a significantly higher risk of irregular menstrual cycles compared to those with early menarche (25). Furthermore, studies have suggested that a shorter gynecological age (calculated as the difference between calendar age and menarcheal age) is strongly inversely associated with the prevalence of irregular menstrual cycles during adolescence (26). In this regard, studies have suggested that the higher incidence of infrequent menstruation and menstrual cycle irregularity among girls who were older at menarche may be primarily explained by their younger gynecological age (27).

In contrast, a study in Debre Berhan found that early menarche was associated with a fourfold increase in the risk of irregular menstruation compared to women who began menstruating at 13–14 years old (10). This discrepancy may be attributed to differences in study setting and the age range of respondents, as fewer women in the mid- and late reproductive age groups (6.3%) participated in that study compared to the present one. Nevertheless, late or delayed menarche remains most commonly associated with AUB, particularly in mid and late adulthood (28).

In addition, the current study found a significant association between a history of abortions and abnormal uterine bleeding (AUB). This finding is consistent with a study conducted in Jimma, which revealed that participants with a history of abortions had more than a 1.5-fold increased risk of AUB (13). Similarly, research from Beijing, China, showed that women who had undergone multiple abortions (≥3) were at a heightened risk for heavy menstrual bleeding (HMB) (29). This association may be attributed to shared underlying causes, such as bleeding disorders, which are common to both abortion and AUB. Studies suggest that 20%–29% of women with HMB have an underlying bleeding disorder (29, 30); however, recognition of these conditions is often challenging, as women frequently overlook their symptoms until significant bleeding events occur. This underscores the need for careful monitoring and early diagnosis in women with a history of abortion.

Anemia, which is the most prevalent condition observed in this study, was found in 13.9% of participants, with a significant association between a history of diagnosed anemia and abnormal uterine bleeding. These findings align with a study conducted in Debre-Berhan, Ethiopia, which also highlighted a strong link between anemia and menstrual irregularities (10). Similarly, research by Rupali A. and Sanjay S. demonstrated that anemic girls are more likely to experience both frequent and infrequent menstrual cycles (31). Furthermore, a study by Panat found that girls with anemia tended to have significantly longer menstrual cycles (32). This growing body of evidence underscores the important connection between anemia and menstrual disturbances, suggesting a need for increased attention to anemia in women with abnormal uterine bleeding.

On the other hand, this study identified a significant association between hypertension and abnormal uterine bleeding (AUB), a finding supported by research conducted in Austria, which also revealed a strong link between chronic hypertension and AUB (33). Similarly, a study in India found that approximately 30.8% of women with AUB also had comorbid hypertension (34). This association may be explained by the fact that high blood pressure can damage blood vessels, including those in the uterus, impairing blood flow to the uterus and ovaries. Such vascular damage can lead to irregular periods, heavy bleeding, or even the absence of menstruation altogether (35).

### Strengths and limitations of the study

The strength of this study lies in its community-based design and the use of the updated 2018 FIGO terminology and definition of abnormal uterine bleeding (AUB), which facilitated a more effective investigation. However, the cross-sectional study design limits the ability to establish a cause-and-effect relationship due to temporality issues. Additionally, recall bias may have been introduced, as women were asked about events occurring within the three months prior to the study. While this limitation is inherent in survey-based research, it is difficult to fully mitigate due to the sensitive and often private nature of menstruation.

## Conclusion and recommendations

This study shows that more than one-third of reproductive-age women in Kombolcha town have experienced abnormal uterine bleeding. Late reproductive age, a history of late menarche, a history of abortion, a history of diagnosed anemia, and hypertension were factors significantly associated with abnormal uterine bleeding.

Healthcare providers should offer adequate medical attention to women with abnormal uterine bleeding in their late reproductive years, as this may indicate underlying diseases that pose a major risk to their health. Additionally, providers should educate patients with abnormal uterine bleeding about relevant lifestyle changes, treatment options, and when to seek emergency care. They should also prioritize the evaluation of anemia in women presenting with AUB. Further longitudinal studies are necessary to better understand the role of risk factors in the cause of AUB and the impact of modifying these factors.

## Data Availability

The original contributions presented in the study are included in the article/Supplementary Material, further inquiries can be directed to the corresponding author.
